# CDK-Mediator and FBXL19 prime developmental genes for activation by promoting atypical regulatory interactions

**DOI:** 10.1093/nar/gkaa064

**Published:** 2020-01-30

**Authors:** Angelika Feldmann, Emilia Dimitrova, Alexander Kenney, Anna Lastuvkova, Robert J Klose

**Affiliations:** Department of Biochemistry, University of Oxford, South Parks Road, Oxford OX1 3QU, UK

## Abstract

Appropriate developmental gene regulation relies on the capacity of gene promoters to integrate inputs from distal regulatory elements, yet how this is achieved remains poorly understood. In embryonic stem cells (ESCs), a subset of silent developmental gene promoters are primed for activation by FBXL19, a CpG island binding protein, through its capacity to recruit CDK-Mediator. How mechanistically these proteins function together to prime genes for activation during differentiation is unknown. Here we discover that in mouse ESCs FBXL19 and CDK-Mediator support long-range interactions between silent gene promoters that rely on FBXL19 for their induction during differentiation and gene regulatory elements. During gene induction, these distal regulatory elements behave in an atypical manner, in that the majority do not acquire histone H3 lysine 27 acetylation and no longer interact with their target gene promoter following gene activation. Despite these atypical features, we demonstrate by targeted deletions that these distal elements are required for appropriate gene induction during differentiation. Together these discoveries demonstrate that CpG-island associated gene promoters can prime genes for activation by communicating with atypical distal gene regulatory elements to achieve appropriate gene expression.

## INTRODUCTION

Multicellular organism development requires accurate spatio-temporal control of gene expression. To achieve this, gene promoters must integrate gene regulatory inputs in order to create appropriate transcriptional outputs. This is controlled by transcription factors that bind to gene regulatory elements, which are often located at large distances from gene promoters, in some cases several hundred of kilobases away from their target gene (1–4). Therefore, it has been proposed that these elements must communicate to achieve appropriate gene expression. In many cases this is thought to rely on direct physical contacts between distal regulatory elements and their target gene promoters (5–7). However, the molecular mechanisms that underpin these physical interactions remain poorly understood.

The Mediator complex is a central regulator of RNA polymerase II (RNAPolII)-dependent gene expression (8). Mediator can interact directly with both transcription factors, which often bind to distal regulatory elements and promoter-bound RNAPolII (9–12). This is thought to enable Mediator to bridge promoters and enhancers (13,14). An alternative form of the Mediator complex, known as CDK-Mediator, contains a kinase module composed of CyclinC, CDK8/19, MED13/13L and MED12/12L. The kinase module binds to the Mediator holocomplex in a manner that is mutually exclusive with RNAPolII, suggesting that Mediator may have roles distinct from directly regulating RNAPolII (reviewed in (15)). Indeed, there is evidence that CDK-Mediator contributes to gene induction in mammals (16–19). Interestingly, the CDK-Mediator complex has also been proposed to support interactions between distal regulatory elements and gene promoters, despite its inability to interact with RNAPolII (20–22). This suggests that there may be alternative, transcription-independent, mechanisms which allow CDK-Mediator at distal regulatory elements to interact with gene promoters.

In mammals, the majority of gene promoters reside within DNA elements that have a high density of non-methylated CpG dinucleotides, called CpG islands (23). These are bound by a family of proteins containing a zinc finger (ZF)-CxxC domain that recognizes non-methylated CpGs. ZF-CxxC domain containing proteins are thought to regulate the activity of RNAPolII through their effects on chromatin at gene promoters (23). However, we recently discovered that a ZF-CxxC domain containing protein, called FBXL19, binds to CpG islands, but is not associated with chromatin-modifying activity (24). Instead, FBXL19 interacts with, and plays a role in targeting, the CDK-Mediator complex to non-transcribed developmental gene promoters in mouse embryonic stem cells (ESCs). If either FBXL19 or CDK-Mediator is removed prior to induction of differentiation, a subset of these developmental genes fail to be properly induced, suggesting that FBXL19 primes these genes for activation via CDK-Mediator. However, the mechanisms that underpin this priming effect remain unknown.

CDK-Mediator has been proposed to support promoter-distal regulatory element interactions and FBXL19 can recruit CDK-Mediator to CpG island-associated gene promoters. Therefore, we hypothesized that FBXL19 may use CDK-Mediator to link distal regulatory elements to promoters and prime genes for future activation. To test this, we used chromosome conformation capture-based approaches and discovered that FBXL19 enables long-range interactions between the CpG island promoters it binds to and other regions of the genome (‘distal sites’) in ESCs. These interactions rely on CDK-Mediator but do not persist (25,26) when genes are activated during differentiation, unlike other typical distal regulatory element interactions (25,26). Interestingly, these distal sites have low levels of histone H3 lysine 27 acetylation (H3K27ac), a histone modification associated with transcriptional activity, both before and after gene induction, further supporting the atypical nature of these interactions. Nevertheless, we show that for the genes tested, these distal sites are required for appropriate gene induction during differentiation, indicating that they function as distal gene regulatory elements. Therefore, FBXL19-dependent recruitment of CDK-Mediator to CpG islands of silent developmental gene promoters helps to support long-range interactions with regulatory elements, thereby priming genes for appropriate activation during differentiation.

## MATERIALS AND METHODS

### Experimental

#### Capture-C library preparation

Capture-C libraries were prepared as described previously (27) with 4 (ESC *Fbxl9-CxxC^fl/fl^*), 3 (*Med13/13l^fl/fl^*) and 2 (RA *Fbxl9-CxxC^fl/fl^*) biological replicates. Briefly, 10 million mouse ES cells were trypsinized, collected in 50 ml falcon tubes in 9.3 ml media and crosslinked with 1.25 ml 16% formaldehyde for 10 min at room temperature. Cells were quenched with 1.5 M glycine, washed with phosphate-buffered saline and lysed for 20 min at 4°C lysis buffer (10 mM Tris pH 8, 10 mM NaCl, 0.2% NP-40, supplemented with Complete proteinase inhibitors, Roche) prior to snap freezing in 1 ml lysis buffer at −80°C. Lysates were then thawed on ice, pelleted and resuspended in 1 ml water prior to being homogenized in a 1 ml dounce homogeniser. Nuclei were checked under the microscope prior to being pelletted again and resuspended in 650 μl of water. Three 1.5 ml tubes with 200 μl lysate each were treated in parallel with sodium dodecyl sulphate (0.28% final concentration, 1 h, 37°C, shaking at 700 rpm), quenched with trypsin (1.67% final concentration, 1 h, 37°C, shaking at 700 rpm) and subjected to a 26 h digestion with 3 × 10 μl DpnII (homemade, 37°C, shaking at 700 rpm). Each chromatin aliquot was independently ligated with 8 μl T4 Ligase (240U) in a volume of 1440 μl (20 h, 16°C). Following this, chromatin was reverse-crosslinked, RNAse H treated and the ligated DNA was phenol–chloroform purified. The sample was resuspended in 300 μl water and sonicated 13× (Pico Bioruptor, 30 s on/30 s off) or until a fragment size of ∼200 bp was reached. Fragments were size selected using AmpureX beads (Beckman Coulter: A63881, ratios: 0.85×/0.4×). 2 × 1–5 μg of DNA were adaptor ligated and indexed using the NEBNext DNA library Prep Reagent Set (New England Biolabs: E6040S/L) and NEBNext Multiplex Oligos for Illumina Primer sets 1 (New England: E7335S/L) and 2 (New England: E7500S/L). The libraries were amplified 7x using Herculase II Fusion Polymerase kit (Agilent: 600677).

#### Capture-C hybridization and sequencing

The probes described in Supplementary Table S1 were pooled at 2.9 nM each. Samples were captured twice and hybridizations were carried out for 72 h and for 24 h for the first and the second captures, respectively. To even out capture differences between tubes, libraries were pooled prior to hybridization at 1.5 μg each. Hybridization was carried out using Nimblegen SeqCap (Roche, Nimblegen SeqCap EZ HE-oligo kit A, Nimblegen SeqCap EZ HE-oligo kit B, Nimblegen SeqCap EZ Accessory kit v2, Nimblegen SeqCap EZ Hybridization and wash kit) following manufacturer's instructions. The captured library molarity was quantified by quantitative polymerase chain reaction (qPCR) using SensiMix SYBR (Bioline, UK) and KAPA Illumina DNA standards (Roche) and sequenced on Illumina NextSeq 500 platform for 4 (ESC *Fbxl19-CxxC^f/f^*), 3 (*Med13/13l^f/f^*) or 2 (RA *Fbxl19-CxxC^f/f^*) biological replicates.

#### (Calibrated) Native ChIP-sequencing and ATAC-sequencing

cChIP-seq for H3K27ac (rabbit anti-mouse-H3K27ac, Cell Signaling, Cat# 8173) and native ChIP-seq for H3K4me1 (rabbit anti-mouse-H3K4me1, Cell Signaling, Cat# 5326) were performed as previously described in (28) in biological duplicates (H3K27ac) or quadruplicates (H3K4me1). ChIP-seq libraries were sequenced on Illumina NextSeq500 using 40 bp paired-end reads.

#### ATAC-seq

ATAC-seq was performed according to (29) in biological quadruplicates. ATAC-seq libraries were sequenced on Illumina NextSeq500 using 80 bp paired-end reads.

#### Cell culture

ESC culture was performed as described previously (30). Tamoxifen and retinoic acid (RA) treatments were performed as described in (24).

#### CRISPR editing

In order to delete putative gene regulatory elements, we generated sgRNAs using the CRISPOR online tool (http://crispor.tefor.net/crispor.py) immediately flanking the summits of the promoter-interacting sites. Sequences are indicated in Table 1. Transfection and clonal selection were carried out as previously described (28). Clones were then screened for deletions by PCR with primers flanking the targeted sites for *Fli1* and *Hoxb3* enhancers (Fli1E FP: CTCGCTCCGGTTCTCCTTTC, Fli1E RP: AATTGGGGAGTGGGTGTGTG, Hoxb3E FP: TGAGGGTGGGGATGTCAAAC, Hoxb3E RP: CGGGTTGGAGGAAGGACAAA). To ensure homozygous deletions, *Fli1E* clones for which a deleted allele was detected were additionally screened for wild-type alleles using PCR primers flanking the 5′ targeting site (Fli1E FP: AGTCTAGCCGCCACTTTTCC, Fli1E RP: GCTGTTTTGGCCCTTTCTGG).

**Table 1.** sgRNAs sequences

Deleted region	sense	antisense
Fli1 enhancer, 5′ end	CACCGAGCCGTGCGCTCCCGAGGTG	AAACCACCTCGGGAGCGCACGGCTC
Fli1 enhancer, 3′ end	CACCGACCGACCCCGACGACCGCAG	AAACCTGCGGTCGTCGGGGTCGGTC
Hoxb3 enhancer, 5′ end	CACCGCTCAACCTTAGGGCCACTCC	AAACGGAGTGGCCCTAAGGTTGAGC
Hoxb3 enhancer, 3′ end	CACCGCGGGTTACTAGCCCCTCCGA	AAACTCGGAGGGGCTAGTAACCCGC

#### Passaging and differentiation of CRISPR clones

The 4 (*Fli1E* deletion) and 3 (*Hoxb3E* deletion) clones were identified and differentiated using RA side-by-side with 2 (*Fli1E* deletion) or 4 (*Hoxb3E* deletion) wild-type clones isolated during the same experiment to obtain true biological replicates. The experiment was repeated 3 (*Fli1E* deletion) and 2 (*Hoxb3E* deletion) times.

#### Reverse transcription and gene expression analysis

Total RNA was isolated from ESCs or RA-treated ESCs using either TRIzol reagent (Thermo scientific) for *Fli1* enhancer deletions or the RNeasy Miniprep kit (Qiagen) for the *Hoxb3* enhancer deletion and *Med13/13l^fl/fl^* cells following manufacturer's instructions and cDNA was synthesized from 400 ng RNA using random primers and ImProm-II Reverse Transcription system kit (Promega). RT-qPCR was performed using SensiMix SYBR mix (Bioline) with the primers indicated in Table 2. *Ikbkap* and *Bcor* genes were used as controls for *Fli1E* and *Hoxb3E* deletions, respectively and *Rad23b* was used for *Med13/13l^fl/fl^* cells. For each clone mean expression levels across 3 (*Fli1E* deletion) or 2 (*Hoxb3E* deletion) technical replicates were calculated. Of these means the mean expression level was calculated across all clones for deletion and corresponding wild-type clones. The expression levels were then normalized to wild-type.

**Table 2.** qRT-PCR Primer sequences

Primer	Sequence
Ikbkap FP	GCTGGCGGTATCTATGCTGT
Ikbkap RP	TCTGCCGAAAGACTGTCACC
Rad23b FP	TAATTGCAGCCCTGAGAGCC
Rad23b RP	TAGTTGCTGTCGTGGTTGCT
Hoxb3 FP	CCAACTCCACCCTCACCAAA
Hoxb3 RP	GCCACCACCACAACCTTCT
Fli1 FP	CAACCAGCCAGTGAGAGTCA
Fli1 RP	GCCCACCAGCTTGTTACATT
BCOR FP	ACTCCGAGGTGTGCAAATTC
BCOR RP	CTGACAGTTTGCGTTTCCTG
Nr2f1 FP	GCCTCAAGAAGTGCCTCAAAG
Nr2f1 RP	GTGCATACTGGCCTGGATTG

#### Immunoblot and antibodies

Nuclear extracts were prepared as previously described (24) and subjected to gel electrophoresis on 3–8% NuPage Tris-Acetate gels (EA0375BOX, ThermoFisher Scientific). A polyclonal antibody against FBXL19 was prepared in-house by rabbit immunization (PTU/BS Scottish National Blood Transfusion Service) (24). Rabbit anti-MED13L (A302-420A, Bethyl laboratories), rabbit anti-MED13 (GTX129674, Genetex) were used to verify conditional deletion of MED13/13l. Rabbit anti-BRG1 (ab110641, Abcam) and rabbit anti-SUZ12 (3737, Cell Signaling) were used for loading controls and anti-rabbit HRP (7074P2, Cell Signaling) as secondary antibody.

### Data analysis

#### Definition of FBXL19-responsive genes

Published 4su-RNA sequencing data were analysed using DESeq2 (31) as previously described (24). Normalized counts were extracted from the DESeq2 table and FPKMs calculated from these. For visualization purposes the normalized counts were log10 transformed upon addition of a pseudocount of 8. Log2 fold changes were extracted from the DESeq2 table.

Genes transcriptionally induced in RA-differentiation were defined as genes with a fold change ≥2 and padj<0.1 between untreated ESCs and RA-treated ESCs. ‘Underinduced genes’ are genes that are normally induced but whose transcriptional levels in RA-differentiated cells are reduced in *Fbxl19-CxxC^fl/fl^* cells treated with tamoxifen (fold change <0.667, padj<0.1). ‘FBXL19-responsive genes’ were defined as underinduced genes whose promoters are occupied by FBXL19. For this, FBXL19-FS2 and FBXL19-deltaCxxC-FS2 ChIP-seq enrichments (24) were quantified within 1 kb of RefSeq TSSs (Refseq transcript table downloaded from UCSC on 21.08.2015). Cutoffs were chosen based on bimodal distributions. Promoters with more than 39.39662 counts (log2 counts >5.3) in the FBXL19-FS2 ChIP-seq and a fold change of more than 1.274561 (log2 >0.35) between FBXL19-FS2 and FBXL19-deltaCxxC-FS2 were defined as FBXL19-bound.

#### Statistical comparison of gene groups

**Figure 1.**
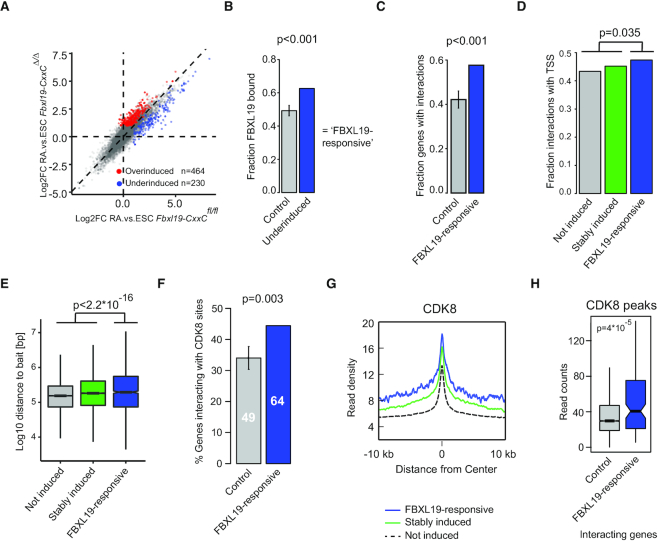
FBXL19-responsive genes interact with distal sites that are bound by CDK8 in ESCs. (**A**) A scatter plot comparing gene expression changes in wild-type (*Fbxl19-CxxC*^fl/fl^, x-axis) and *Fbxl19-CxxC^Δ^^/^^Δ^* (y-axis) cells following RA treatment. The axes correspond to log2 fold changes in 4sU-seq between ESCs and RA-treated cells. Underinduced genes are marked in blue and overinduced genes in red. (**B**) A bar chart illustrating the fraction of underinduced genes (blue) and transcriptionally matched control genes (grey) which are bound by FBXL19. The *P*-value corresponds to 1000 random samplings. (**C**) A bar chart illustrating the fraction of FBXL19-responsive genes (blue) or transcriptionally matched control genes (grey) that interact with any putative regulatory region as defined by ATAC-seq. The *P*-value corresponds to 1000 random samplings. (**D**) A bar chart illustrating the fraction of interactions with ATAC-seq peaks located within 1 kb of an annotated TSS for FBXL19-bound genes that are not induced (grey), induced independently of FBXL19 (Stably Induced, green) and those that rely on FBXL19 for their induction (Responsive, blue). The *P*-value corresponds to 10 000 random samplings. (**E**) A box pot showing the distance between FBXL19-bound promoters of not induced (grey), stably induced (green) and FBXL19-reponsive (blue) and their interacting distal site. The boxplots display the median, 25th and 75th quartile. *P*-value: Student's *t*-test. (**F**) A bar chart showing the percentage of FBXL19-responsive (blue) and transcriptionally matched control (grey) gene promoters that interact with a CDK8-bound site. The *P*-value corresponds to 1000 random samplings. Number of gene promoters is indicated within the charts. (**G**) A metaplot of CDK8 ChIP-seq enrichment (24) centred at ATAC-seq peaks interacting with either not induced (dashed-black), stably induced (green) or FBXL19-responsive (blue) FBXL19-bound gene promoters. (**H**) A boxplot comparing CDK8 levels at CDK8 peaks interacting with either FBXL19-responsive (blue) or other (grey) FBXL19-bound gene promoters. *P*-value: Student's *t*-test.

To define groups of genes with matched transcription in Figure 1, first FPKM from RefSeq transcripts were binned into 10 equally sized bins using the cut2() function from the CRAN package Hmisc. Equal number of control genes were randomly selected to have the same number of genes in each bin as the genes of interest (e.g. FBXL19-responsive genes). Empirical *P*-values were derived based on *n* = 1000 random samplings.

#### Putative regulatory elements

Putative regulatory elements were based on ATAC-seq peak calling from (32). To determine whether they overlapped with a TSS, the widest possible set of annotated TSSs was used by combining UCSC, RefSeq and Ensembl Gene TSSs (downloaded from UCSC on 16.02.2016, 21.08.2015 and 28.08.2015, respectively). TSS was defined as transcription start site ±1000 bp and each regulatory region overlapping this 1 kb region surrounding the TSS was regarded as ‘TSS-overlapping’. ATAC peaks within 100 bp of a CDK8 peak (24) were considered as CDK8-overlapping. ATAC-Peaks interacting with a gene promoter in Capture-HiC were determined as described in ‘Capture-C and Capture-HiC’ analysis.

#### Capture-C and Capture-HiC analysis

For Capture-C, paired-end reads were aligned to mm10 and filtered for HiC artefacts using HiCUP (33) and Bowtie2 (34) with standard settings for published data (35) and with fragment filter set to 100–800 bp for all other datasets. Read counts of reads aligning to captured gene promoters and interaction scores (=significant interactions) were then obtained by using the Bioconductor package CHiCAGO (Chicago_1.6.0, (35)). Individual replicates were visually inspected for quality and high correlation across captured interactions was ensured prior to continuation (see Supplementary Table S3).

For visualization of Capture-C using line plots, weighted pooled read counts from CHiCAGO data tables were normalized to total read count aligning to captured gene promoters and further to the number of promoters in the respective capture experiment. Line plots were plotted from normalized chicago data objects using the runmean() function from the CRAN package caTools (https://CRAN.R-project.org/package=caTools). For visualization in UCSC Genome Browser, bedgraphs were generated from normalized chicago data tables for each promoter in capture and converted to bigwig files using bedGraphToBigWig (36).

For comparative boxplot analysis, interactions called by CHiCAGO (score ≥ 5) across *Fbxl19-CxxC^fl/fl^* and *FBXL19-CxxC^Δ^^/^^Δ^* were aggregated and interactions with a distance of <4 DpnII fragments were merged to a single interaction peak. For each interaction peak we then calculated the sum of normalized read counts and CHiCAGO scores across all overlapping DpnII fragments and normalized this sum by the number of DpnII fragments in each peak. Interactions with a CDK8 peak (24) or a putative regulatory site within 300 bp were considered CDK8-positive or regulatory, respectively. To define gained and lost interactions used in Supplementary Figure S2, we required interactions to have both a higher/lower interaction score and normalized read coverage in *Fbxl19-CxxC^Δ^^/^^Δ^* ESCs in at least three out of four replicates (Supplementary Table S2).

#### Capture-C probe design

Test promoters for Capture-C analysis were selected to examine FBXL19-responsive genes and a complementary set of control gene promoters (24,37). Genes were further preselected based on their interaction with regulatory sites in published genome-wide promoter Capture-HiC (38). To avoid ambiguity in data interpretation, promoters were pre-filtered based on having a unique TSS within the captured HindIII and DpnII fragments and the captured DpnII fragment was required to be larger than 200 bp for probe design. Per captured DpnII fragment 2 5′ biotinylated probes were designed using an online tool by the Hughes lab (CapSequm, http://apps.molbiol.ox.ac.uk/CaptureC/cgi-bin/CapSequm.cgi) to be 70–120 bp long. If no probe design was possible for the fragment overlapping the TSS, probes were designed within the fragment immediately up- or downstream (in this order), provided they did not contain another TSS. This allowed us to generate probes for 36 FBXL19-responsive and 196 control gene promoters. All resulting probes are detailed in Supplementary Table S1.

#### Alignment and processing of ChIP-seq and ATAC-seq data

Datasets were aligned to the mm10 mouse genome (ChIP-seq and ATAC-seq) or to the combined dm6-mm10 drosophila-mouse genome (cChIP-seq) and processed as previously described (28,29). High correlation across replicates (Pearson's correlation coefficient: > = 0.94 for ChIP-seq and > = 0.90 for ATAC-seq) was ensured prior to pooling of normalized samples. BigWig and BedGraph files were generated using MACS2 pileup function (39). ChIP-seq enrichments were quantified from bedgraph files using the annotatePeaks.pl script from HOMER (40) (Figure 4) or from bam files using the function summarizeOverlaps(paired = TRUE) from GenomicFeatures (41) (Supplementary Figure S4, H3K4me1). Log2 transformations were calculated following the addition of a pseudocount of 1.

#### Processing of 4sU-RNA-seq data

4sU-RNA-seq data were processed as previously described (24). To distinguish between gene and eRNA transcription in Supplementary Figure S4, 4sU-tags were quantified within only those sites that did not overlap with an expressed gene (cutoff = 1.1435 FPKM). Quantification was made from bam files using the function summarizeOverlaps(paired = TRUE) from GenomicFeatures (41) (Supplementary Figure S4).

### Datasets

The datasets generated for this study are available in GEO database under the accession number GSE136424. Datasets reanalysed for this study can be found in the GEO database under the accession numbers GSE98756 (FBXL19-FS2 and CDK8 ChIP-seqs as well as 4sU-RNASeq), GSE34520 (PolII ChIP-seq) and in the ArrayExpress database under the accession number E-MTAB-2414 (Capture-HiC).

## RESULTS

### FBXL19-responsive genes interact with distal sites that are bound by CDK8 in ESCs

FBXL19 binds to CpG island-associated gene promoters, interacts with CDK-Mediator and contributes to the activation of developmental genes during differentiation (24). Therefore, we hypothesized that FBXL19 may promote gene activation by linking gene promoters to distal regulatory elements via CDK-Mediator. To test this possibility we first identified a stringent set of genes that require FBXL19 for appropriate activation when ESCs are induced to differentiate with RA (Figure 1A and Supplementary Figure S1A). In agreement with our previous observations that these genes are associated with developmental pathways (24), they are essentially non-transcribed in the ESC state (Supplementary Figure S1B). Approximately 60% of these genes are occupied by FBXL19, and we refer to these as ‘FBXL19-responsive’ genes (Figure 1B). In order to determine whether the promoters of FBXL19-responsive genes interact with other regulatory elements, we examined genome-wide promoter Capture Hi-C from ESCs (38). This method detects interactions that occur between a specific gene promoter and other sites in the genome that are located distally in respect to this promoter, hereafter termed ‘distal sites’. We then identified interactions between FBXL19-responsive gene promoters and transposase accessible (ATAC-seq) regions of the genome (32), which often correspond to gene regulatory elements (both promoters and enhancers) (42). We found that ∼60% of FBXL19-responsive gene promoters interacted with another accessible region of the genome, despite their low transcriptional activity in the ESC state (Figure 1C and Supplementary Figure S1C). Similarly to other types of genes, distal regions that FBXL19-responsive genes interacted with were segregated roughly equally between other TSSs and accessible regions not annotated as a TSS (Figure 1D and Supplementary Figure S1D-E), but tended to span larger distances (Figure 1E and Supplementary Figure S1F).

FBXL19-responsive gene promoters are characterized by high occupancy of CDK-Mediator (24). We were therefore curious to know whether distal sites that these genes interact with also show evidence for CDK-Mediator engagement. To test this possibility we examined CDK8 ChIP-seq (24). Interestingly, we found that FBXL19-responsive promoters interacted frequently with CDK8-occupied sites (Figure 1F) and that these interacting sites had elevated levels of CDK8 (Figure 1G and H), irrespective of whether they overlapped an annotated TSS (Supplementary Figure S1I). These distal sites also were occupied by RNAPolII (43), but not by its elongating form (Supplementary Figure S1G) and displayed little if any enhancer transcript production (Supplementary Figure S1H). Interestingly, they were bound by FBXL19 (Supplementary Figure S1I), and CDK8 occupancy at these sites displayed a very small reduction in its absence (Supplementary Figure S1J). Given that FBXL19 can engage with promoters and some interacting distal sites, it may help to recruit CDK-Mediator to both locations and possibly support their interaction. Together, this indicates that FBXL19-responsive gene promoters interact with distal sites which are also occupied by CDK-Mediator.

### FBXL19 contributes to interactions between FBXL19-responsive gene promoters and CDK8-occupied distal sites

**Figure 2.**
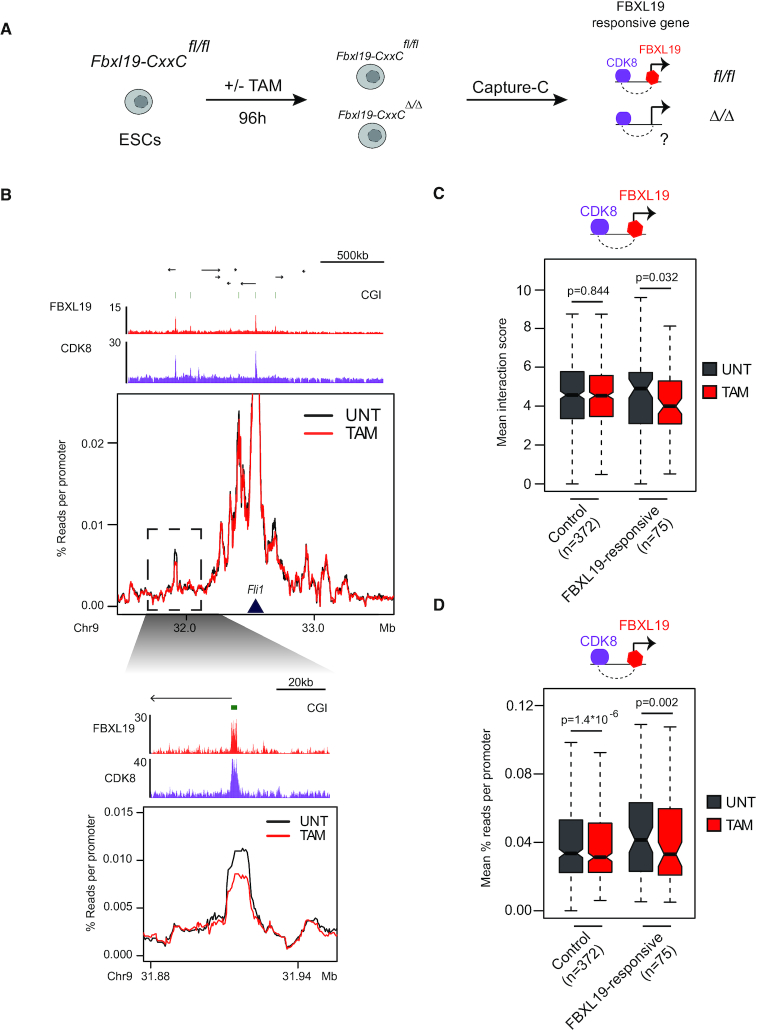
FBXL19 contributes to interactions between FBXL19-responsive gene promoters and CDK8-occupied distal sites. (**A**) A schematic illustrating the workflow of the experiment. (**B**) A comparison between the Capture-C signal in untreated (black) and tamoxifen treated (red) *Fbxl19-CxxC ^fl/fl^* ESCs for the FBXL19-responsive gene *Fli1*. The Capture-C viewpoint is indicated by the dark-blue triangle. Genome browser tracks for FBXL19 and CDK8 ChIP-seq (24) are shown in red and purple, respectively, above the Capture-C signal. The region highlighted by the dashed rectangle is shown as a zoom-in below. (**C**) A boxplot showing the Chicago interaction scores for interactions between CDK8-occupied sites and FBXL19-responsive or control gene promoters. The box plots show mean interaction score between test promoters and CDK8-occupied sites in untreated (UNT) or tamoxifen-treated (TAM) *Fbxl19-CxxC ^fl/fl^* ESCs. The control sites correspond to FBXL19-bound, but not FBXL19-responsive, genes. The number of interactions quantified is indicated below the boxplots. *P*-values: paired t-test. (**D**) As in C, but showing normalized read counts in the Capture-C experiments.

Having identified a set of FBXL19-responsive genes that interact with putative regulatory elements enriched for CDK-Mediator, we set out to determine whether FBXL19 was required for these interactions. To achieve this we performed Capture-C (27) in ESCs and examined 36 FBXL19-responsive genes and a set of control genes. We then examined whether disrupting the binding of FBXL19 to CpG islands affected these interactions by using our ESC line that contains a tamoxifen-responsive form of CRE recombinase and in which the exon encoding the ZF-CxxC domain of Fbxl19 is flanked by *loxP* sites (Figure 2A and Supplementary Figure S2A). Addition of tamoxifen deletes the floxed exon leading to the production of a form of FBXL19 that cannot bind CpG islands, but still interacts with CDK-Mediator (24). Importantly, the CxxC-domain deficient form of the FBXL19 protein does not occupy FBXL19-responsive gene promoters (Supplementary Figure S2B), allowing us to study the effect of FBXL19-occupancy on interactions in this cell line. Interestingly, this revealed that interactions between FBXL19-responsive gene promoters and CDK8-occupied sites displayed a modest reduction in both the average interaction strength (Figure 2C) and read density (Figure 2B and D) following removal of FBXL19, whereas other interactions were not reduced (Figure 2B–D and Supplementary Figure S2C–F). This specificity was further evident when we compared interactions that were reproducibly lost or gained in the absence of FBXL19 (Supplementary Figure S2G), with lost interactions being enriched for FBXL19-responsive genes and CDK8-occupied sites (Supplementary Figure S2H). Therefore, FBXL19 contributes to interactions between FBXL19-responsive gene promoters and distal sites that are occupied by CDK8, despite the associated gene being inactive.

### FBXL19-responsive gene interactions require CDK-Mediator

**Figure 3.**
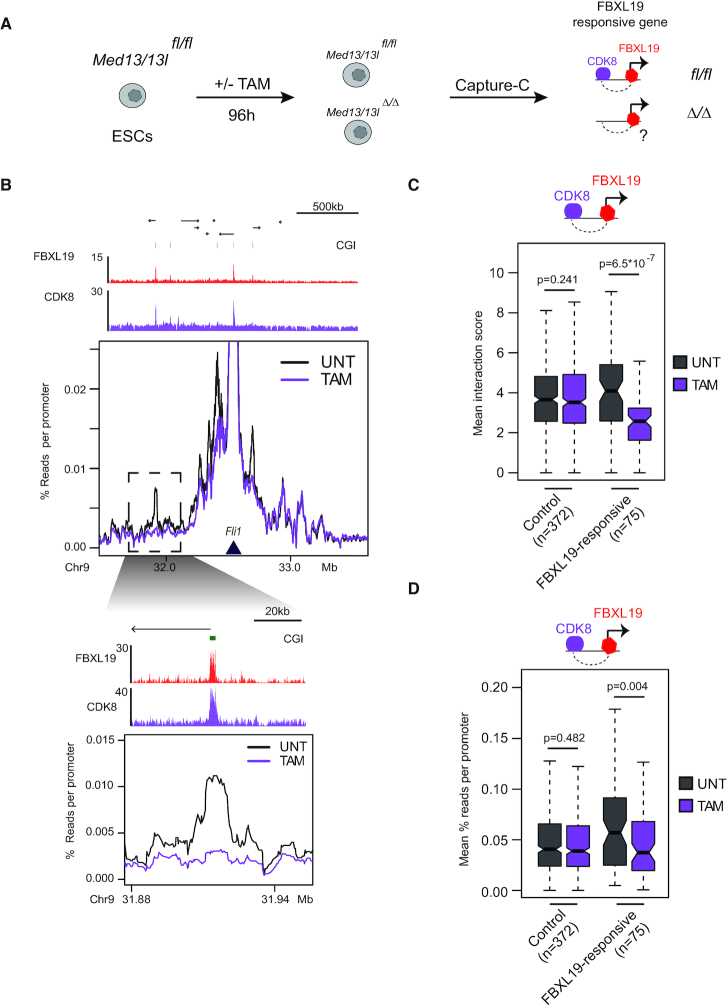
FBXL19-responsive gene interactions require CDK-Mediator (**A**) A schematic illustrating the workflow of the experiment. (**B**) A comparison between the Capture-C signal in untreated (black) and tamoxifen treated (purple) *Med13/13l ^fl/fl^* ESCs for the FBXL19-responsive gene *Fli1*. The Capture-C viewpoint is indicated by the dark-blue triangle. Genome browser tracks for FBXL19 and CDK8 ChIP-seq (24) are shown in red and purple, respectively, above the Capture-C signal. The region highlighted by the dashed rectangle is shown as a zoom-in below. (**C**) A boxplot showing the Chicago interaction scores for interactions between CDK8-occupied sites and FBXL19-responsive or control gene promoters. The boxplots show mean interaction score between test promoters and CDK8-occupied sites in untreated (UNT) or tamoxifen-treated (TAM) *Med13/13l ^fl/fl^* ESCs. The control sites correspond to FBXL19-bound, but not FBXL19-responsive, genes. The number of interactions quantified is indicated below the boxplots. *P*-values: paired *t*-test. (**D**) As in C, but showing normalized read counts in the Capture-C experiments.

FBXL19-responsive genes rely on FBXL19 for interactions with distal sites which also show occupancy by CDK8. This raised the interesting possibility that FBXL19 may work with CDK-Mediator to link these distal sites to FBXL19-responsive gene promoters. To test this possibility we took advantage of an ESC line in which the *Med13* and *Med13l* subunits of Mediator can be conditionally deleted. Importantly, removal of MED13/13L disrupts binding of the CDK-Mediator module to the core Mediator and causes a loss of CDK8 binding to chromatin (Figure 3A and Supplementary Figure S3A-B (24)). Conditional deletion of *Med13/13l* resulted in a strong and specific reduction in interactions between FBXL19-responsive promoters and CDK8-occupied distal sites in the genome (Figure 3B–D and Supplementary Figure S3C–F). We note that the reductions in interactions following CDK-Mediator removal were more pronounced than those observed when FBXL19 DNA binding was perturbed (Figure 2). We have previously shown that FBXL19 contributes to, but does not solely define, the recruitment of CDK-Mediator to developmental gene promoters (24). This may explain why interactions are more modestly affected when the DNA binding of FBXL19 is perturbed (see ‘Discussion’ section). Therefore, FBXL19 and CDK-Mediator appear to support interactions between FBXL19-reponsive gene promoters and CDK8-occupied distal sites.

### The majority of interactions associated with FBXL19-responsive genes do not acquire H3K27ac and are lost during differentiation

**Figure 4.**
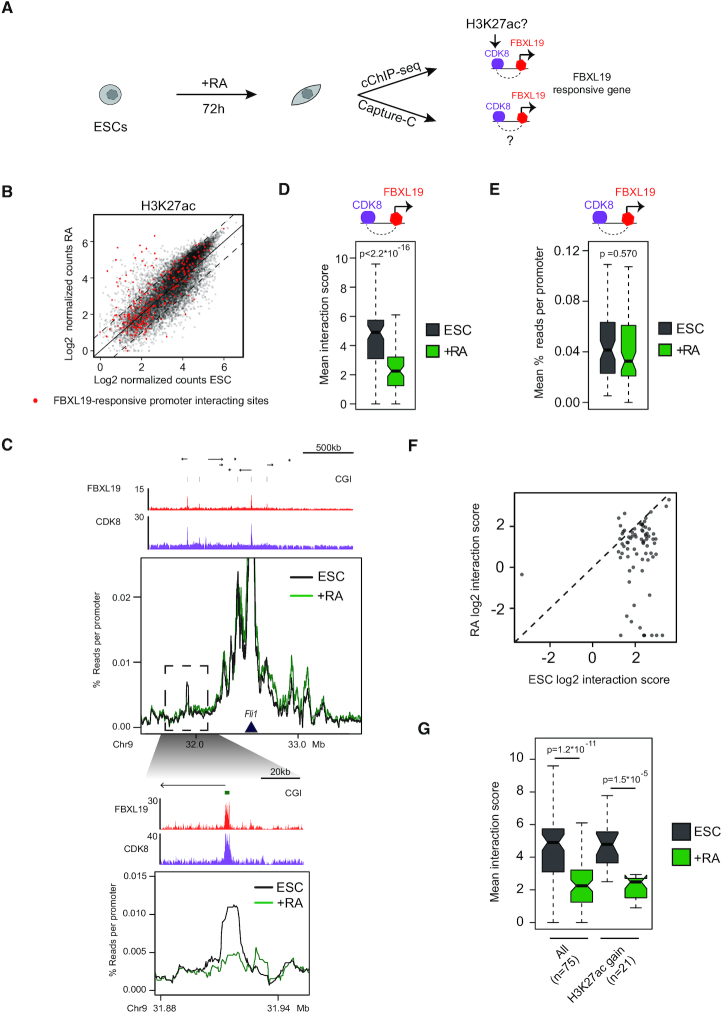
The majority of interactions associated with FBXL19-responsive genes do not acquire H3K27ac and are lost during differentiation (**A**) A schematic illustrating the workflow of the experiment. (**B**) A scatterplot comparing H3K27ac enrichment at FBXL19-occupied gene promoter interacting distal sites (4) which are CDK8-occupied and accessible by ATAC-seq in untreated or RA-treated ESCs (32) (24). FBXL19-responsive gene promoter interacting distal sites are highlighted in red. The dashed lines indicate the 2-fold cut-off applied to determine whether there was a gain or loss of H3K27ac and the solid line corresponds to the diagonal. (**C**) A comparison between the Capture-C signal in untreated (black) and RA-treated (green) ESCs for the FBXL19-responsive gene *Fli1*. The Capture-C viewpoint is indicated by the dark-blue triangle. Genome browser tracks for FBXL19 and CDK8 ChIP-seq (24) are shown in red and purple, respectively, above the Capture-C signal. The region highlighted by the dashed rectangle is shown as a zoom-in below. (**D**) Chicago interaction scores for ESC interactions between Fbxl19-responsive gene promoters and CDK8-occupied sites for untreated (dark grey) and RA-treated (green) ESCs. *P*-value: paired *t*-test. (**E**) As in D but for read counts. Whilst the trend is the same as in D, these effects did not reach significance thresholds (**F**) A scatterplot comparing log2 mean interaction scores between untreated and RA-treated ESCs. (**G**) Chicago interaction scores for interactions between FBXL19-responsive genes and CDK8-occupied sites in the untreated (ESC) and RA-treated cells. The mean interaction scores are shown for interactions with any site (All) or sites that showed increases in H3K27ac upon RA-differentiation (H3K27ac gain). The number of interactions assessed is indicated below the plots. *P*-values: Student's *t*-test.

FBXL19 and CDK-Mediator promote interactions between silent developmental gene promoters and CDK8-bound distal sites. Therefore, we reasoned that these distal sites could act as regulatory elements that contribute to the activation of FBXL19-responsive genes during differentiation. Often, when distal regulatory elements are involved in driving transcriptional activity, they acquire histone H3 lysine 27 acetylation (44). Therefore, we were keen to examine whether distal sites that FBXL19-responsive gene promoters interact with have, or acquire, H3K27ac during differentiation when their associated gene is transcribed. To achieve this we carried out calibrated H3K27ac ChIP-seq before and after RA treatment (Figure 4A). In the ESC state, these putative regulatory sites displayed low levels of acetylation (Supplementary Figure S4A), in agreement with their associated genes lacking transcriptional activity and following RA induced differentiation the majority of sites (81%) showed no evidence for increased acetylation (Figure 4B and Supplementary Figure S4A-B). To further characterize the distal sites that interact with FBXL19-responsive gene promoters, we carried out H3K4me1 ChIP-seq and ATAC-seq in ESCs and following RA treatment. This revealed that the distal sites that interact with FBXL19-responsive gene promoters are accessible yet display lower levels of H3K4me1 compared to active enhancers in ESCs. Following RA treatment these distal sites displayed a reduction in H3K4me1 but their accessibility remained largely unchanged (Supplementary Figure S4A and B).

Interactions between FBXL19-responsive gene promoters and CDK-Mediator bound distal sites were evident in the ESC state even when the respective gene was not transcribed. Previously, interactions involving inactive gene promoters and regulatory elements required for their activation have been reported in several contexts, and these interactions were often maintained following gene activation (2,25–26,45–46). Therefore, we next asked what happens to FBXL19-responsive gene interactions and distal sites during differentiation-induced gene activation. To address this we carried out Capture-C in the ESC state and following RA-induced differentiation (Figure 4A). Interestingly, we observed a near complete and uniform loss of interactions between FBXL19-responsive genes and their associated distal sites (Figure 4C–F), irrespective of whether these sites gained H3K27ac during gene induction (Figure 4G and Supplementary Figure S4C). In contrast, other genes whose induction did not rely on FBXL19, retained interactions with distal sites but only with those that acquired H3K27ac upon differentiation (Supplementary Figure S4D). Together these observations suggest that interactions between FBXL19-responsive gene promoters and distal sites occupied by CDK-Mediator, which may constitute putative distal regulatory elements, have fundamentally different properties from previously described distal regulatory element-promoter interactions (25,26).

### FBXL19-responsive gene promoter-interacting sites are required for gene activation during differentiation

**Figure 5.**
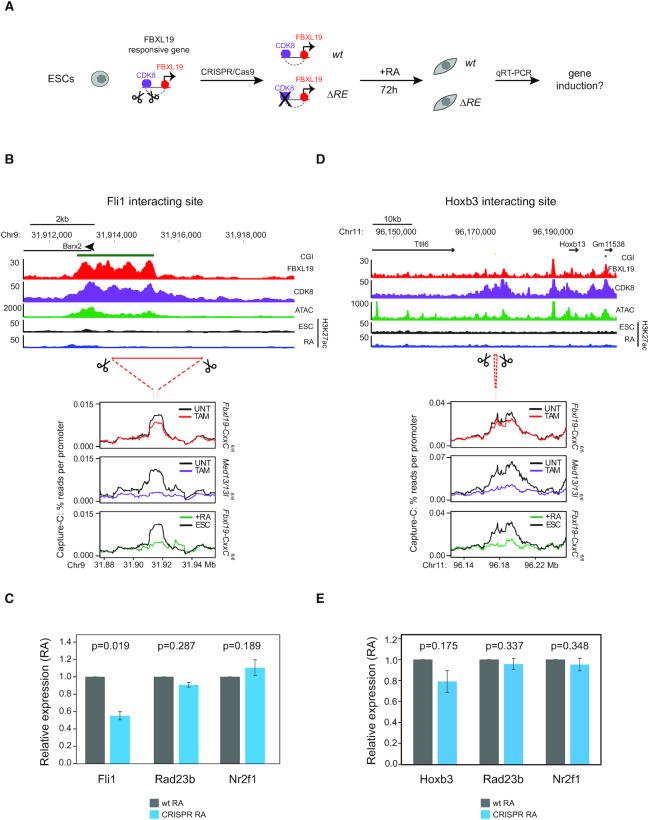
FBXL19-responsive gene promoter-interacting sites are required for gene activation during differentiation (**A**) A schematic illustrating the workflow of the experiment. (**B** and**D**) A screenshot showing the distal interaction site for *Fli1* (B) and *Hoxb3* (D). To highlight the properties of deleted regions, FBXL19, CDK8 and H3K27ac ChIP-seq as well as accessibility (ATAC-seq) are illustrated for each locus. The regions deleted by CRISPR/CAS9 based editing are illustrated with scissors. Below the deleted region the line plots depict the effect on Capture-C signal when FBXL19 or MED13/13L are removed, or when cells are treated with RA. (**C**) Relative mRNA-levels for *Fli1*, *Rad23b* and *Nr2f1* in RA-treated wild-type ESC clones (grey, *n* = 2) and in ESC clones in which the *Fli1* promoter interacting site has been deleted (blue, *n* = 4) as determined by RT-qPCR. Shown is the mean signal normalized internally to a control gene and to expression in wild-type clones treated by RA. Error bars indicate standard error of mean across four biological and three technical replicates. *P*-values: Student's *t*-test. (**E**) Relative mRNA-levels of *Hoxb3*, *Rad23b* and *Nr2f1* in RA-treated wild-type ESC clones (grey, *n* = 4) and in ESC clones in which the *Hoxb3* promoter interacting site has been deleted (blue, *n* = 3) as determined by RT-qPCR. Shown is the mean signal normalized internally to a control gene and to expression in wild-type clones treated by RA. Error bars indicate standard error of mean across three biological and two technical replicates. *P*-values: Student's *t*-test.

The interactions between FBXL19-responsive genes and their putative distal regulatory sites have an atypical combination of features in that they are present prior to gene activation, are lost when transcription is induced during differentiation, and show little evidence for acquisition of H3K27ac, which is often associated with active distal regulatory elements (44). This suggests that the interactions between FBXL19-responsive gene promoters and CDK-Mediator occupied sites may function through regulatory mechanisms that are distinct from other previously characterized pathways, or, perhaps more simply, that these interactions are not actually involved in gene activation. To distinguish between these two possibilities, we focussed on one of these atypical interaction sites associated with the FBXL19-responsive gene *Fli1* which, like the majority of FBXL19-responsive promoter interacting sites, lacked H3K27ac and no longer interacted with the *Fli1* gene promoter following differentiation (Figure 5B). Importantly, similarly to other developmental genes (24), appropriate induction of *Fli1* also relied on CDK-Mediator (Supplementary Figure S5A).To examine what effect the loss of this interaction site had on *Fli1* induction during differentiation, we used CRISPR-Cas9-based genome editing to delete it (Figure 5A). We isolated four independent ESC clones with homozygous deletions (Supplementary Figure S5B and C) and quantified *Fli1* gene expression in ESCs and upon RA treatment. This revealed that deleting this interaction site reduced *Fli1* gene induction by ∼45% during differentiation, whereas expression of a control gene (*Rad23b*) and an unlinked FBXL19-responsive gene (*Nr2f1*) remained unaffected (Figure 5C). Importantly, distal site deletion did not alter gene expression in ESCs prior to differentiation (Supplementary Figure S5D). We then used the same approach to remove a similar atypical site that interacted with the promoter of *Hoxb3*, another gene whose induction depended on CDK-Mediator (Supplementary Figure S5A). Here we observed a modest (∼20%) reduction in gene induction (Figure 5D-E and Supplementary Figure S5E–G). This effect on induction was more limited in magnitude than observed for *Fli1* and the difference did not prove to be statistically significant, consistent with the fact that *Hoxb3* forms several other FBXL19/CDK-Mediator dependent interactions elsewhere in the locus that may also contribute to gene activation (Figure 5D). Nevertheless, together these genome manipulations demonstrate that atypical FBXL19-reponsive promoter interactions can indeed be leveraged to support appropriate gene induction during differentiation. Furthermore, it suggests that FBLX19 and CDK-Mediator prime a subset of non-transcribed genes in ESCs for future activation during development through supporting their communication with distal gene regulatory elements.

## DISCUSSION

We previously demonstrated that the CpG island binding protein, FBXL19, physically interacts with and recruits CDK-Mediator to silent developmental gene promoters in the ESC state to prime them for appropriate activation during differentiation (24). However, the mechanisms that underpin how FBXL19 and CDK-Mediator create this priming effect were unclear. Here we provide evidence that FBXL19-responsive gene promoters in ESCs, despite their lack of transcription, physically interact with distal sites in the genome that also show occupancy of CDK-Mediator (Figure 1). These interactions are supported by FBXL19 and rely on CDK-Mediator, demonstrating that these factors may cooperate in supporting long-range interactions between CpG island-associated promoters and distal sites in the genome independently of transcription (Figures 2 and 3). Interestingly, following induction of differentiation and activation of FBXL19-responsive genes, distal sites that interact with FBXL19-responsive gene promoters in the ESC state generally do not acquire H3K27ac and no longer interact with the promoter (Figure 4). Nevertheless, these distal sites appear to be required for gene induction, as their deletion, much like removal of FBXL19 or CDK-Mediator, causes defects in gene activation for the genes we have examined (Figure 5). We currently focused our deletion analysis on two atypical distal sites that interact with FBXL19-responsive genes. In future work, high throughput perturbation of distal sites that interact with FBXL19-responsive gene promoters will be required to determine the generality of these observations. Together, this provides new mechanistic evidence that FBXL19 and CDK-Mediator can prime genes for activation during differentiation by promoting CpG island-associated gene promoter interactions with distal regulatory elements.

The mechanisms that support the interactions between promoters and distal regulatory elements are beginning to emerge, but these have mostly been studied in the context of actively transcribed genes, where the transcriptional machinery (47), other promoter-bound factors (48,49) or general architectural proteins (20,22) are important for forming interactions. In contrast, the mechanisms supporting interactions between silent genes and their regulatory elements are less well understood, but have been attributed to RNAPolII (26) or chromatin modifying proteins (25,50–52). Here were uncover a distinct situation where FBXL19 and CDK-Mediator appear to play a central role in supporting interactions between silent gene promoters and distal gene regulatory elements in ESCs. This then raises the interesting question of how FBXL19 and CDK-Mediator could mechanistically promote these transcription-independent interactions. One possibility is that FBXL19 may help to recruit CDK-Mediator to silent CpG island-associated gene promoters via its capacity to bind non-methylated CpG dinucleotides. If the interacting regulatory element houses a site-specific DNA binding factor that also interacts with the CDK-Mediator complex (9), this could allow the creation of a tripartite interaction that links the promoter and distal site via a CDK-Mediator bridging event. Alternatively, FBXL19 may help to guide CDK-Mediator to both the promoter and its interacting distal site, physically bridging these elements thought CDK-Mediator. In agreement with this possibility, FBXL19 binding was evident at the promoter of FBXL19-responive genes and also at the distal regulatory elements with which they interact. Furthermore, a small reduction in CDK-Mediator binding occurred at both of these regions when FBXL19 binding was disrupted (Supplementary Figure S1). Inevitably, the precise mechanisms at play are likely more complex, since removal of FBXL19 does not cause as severe defects in gene activation or long-range interactions as removal of CDK-Mediator. This suggests that additional CDK-Mediator dependent mechanisms may also support the observed interactions (53–55). In future work it will be important to further dissect the biochemistry underlying the capacity of FBXL19 and CDK-Mediator to support these transcription-independent promoter distal regulatory element interactions.

The interactions we observe between silent FBXL19-responsive gene promoters and distal regulatory elements appear to function during gene activation in a manner which is distinct from previously characterized gene promoter interactions. Firstly, they are not maintained upon differentiation-induced gene activation as is the case for other stimuli-induced interactions (46), developmental genes (25), or interactions described in other developmental model systems (2,45). Secondly, they do not appear to rely on H3K27ac to support their function during gene activation (Figures 4 and 5). These unique features of FBXL19-responsive gene promoter interactions suggest they may act through distinct mechanisms to prime genes for activation during differentiation. One possibility for how these interactions support gene activation could be that FBXL19 and CDK-Mediator simply function as a physical bridge to hold the promoter and its associated distal regulatory element together in anticipation of an instructive activation signal, possibly the recruitment of a DNA binding transcription activator, which then transitions the promoter into an activated state (25). Following this switch, interactions with the regulatory element may no longer be required for continued gene expression (1). However, given the fact that Mediator is known to play important roles in gene expression, it seems unlikely that its sole role in this activation process is to function as a molecular bridge between the promoter and its distal regulatory site. It is thus possible that its presence also promotes an environment or a concentration of Mediator that favours gene transcription when the appropriate activation signals are present (56).

Taken together we present new evidence that FBXL19 and CDK-Mediator specifically promote interactions between silent developmental gene promoters and distal regulatory elements in ESCs. These interactions prime a subset of these genes for activation during differentiation in a manner that does not rely on acquisition of H3K27ac at the distal site nor on a persistent interaction, which differs from previously characterized developmental promoter interactions. Therefore, this provides a new mechanism for how CpG islands can function to shape long-range chromosomal interactions and gene expression.

## DATA AVAILABILITY

The datasets generated for this study are available in GEO database under the accession number GSE136424.

## Supplementary Material

gkaa064_Supplemental_Files
