# Supplementary material for: CDK-Mediator and FBXL19 prime developmental genes for activation by promoting atypical regulatory interactions
Source: Nucleic Acids Res. 2020 Jan 30;48(6):2942–55. doi: 10.1093/nar/gkaa064 (PMC7102981; doi:10.1093/nar/gkaa064)
Supplement: gkaa064_Supplemental_Files [file gkaa064_supplemental_files.zip › Supplemental Material r2.pdf]

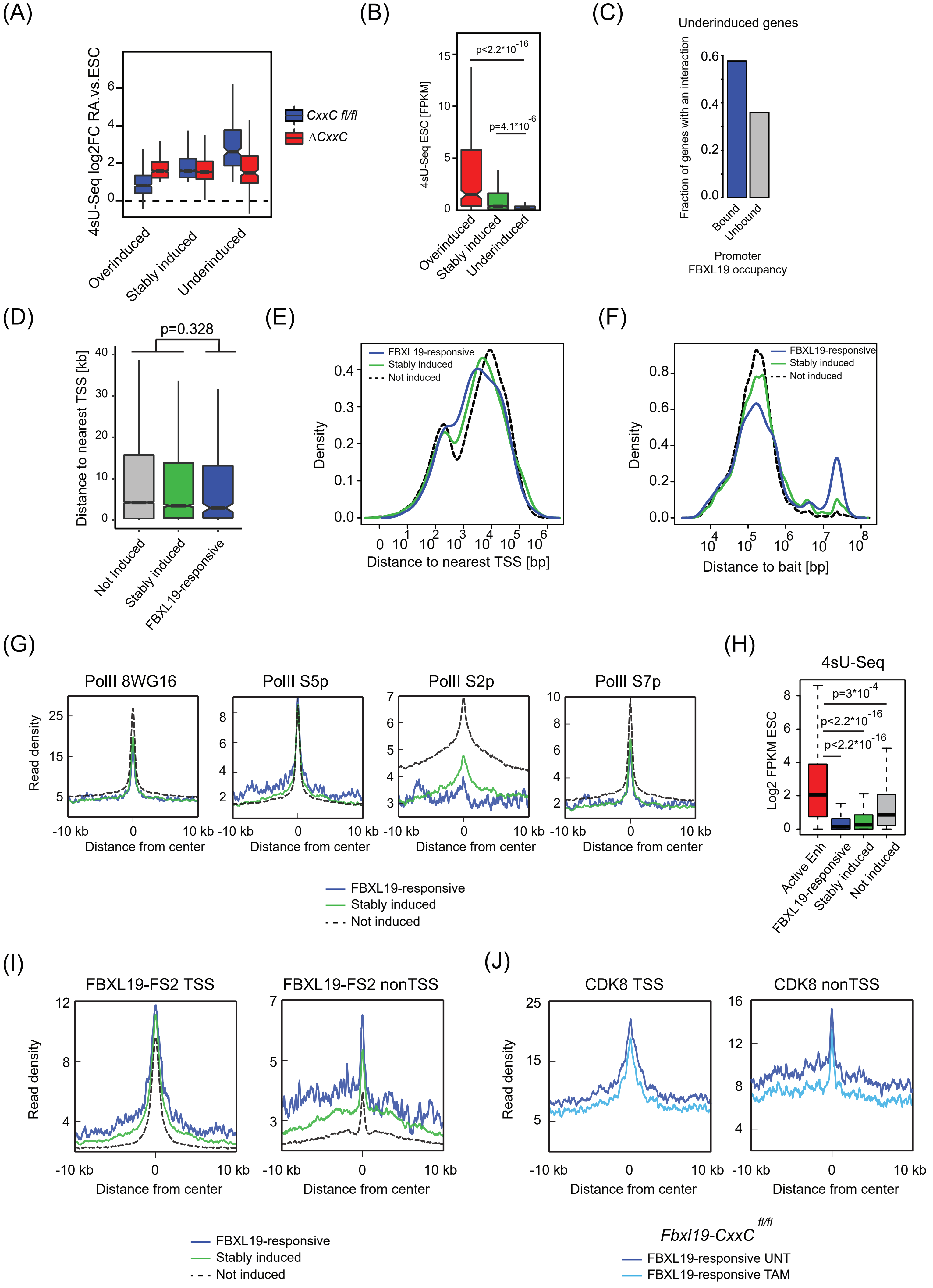

Feldmann et al_Fig. S1

Figure S1. Related to Figure 1

(A) A boxplot comparing gene expression changes for overinduced genes, genes that are stably induced in the absence of FBXL19 and underinduced genes in wild type (*Fbxl19-CxxC*$^{fl/fl}$, blue) and *Fbxl19-CxxC*$^{\Delta/\Delta}$ (red) cells following retinoic acid treatment. The y-axis corresponds to log2 fold changes in 4sU-seq between ESCs and RA-treated cells. The boxplots display the median, 25th and 75th quartile.

(B) A boxplot comparing gene expression levels in ESCs for overinduced genes, genes that are stably induced in the absence of FBXL19 and underinduced genes. The boxplots display the median, 25th and 75th quartile. P-values: Student's t-test.

(C) A bar chart illustrating the fraction of underinduced genes that form interactions with an ATAC-seq peak for FBXL19-bound (blue) or unbound (grey) genes.

(D) A boxplot comparing distances to nearest TSS for ATAC-seq peaks interacting with FBXL19-bound genes that are not induced (grey), induced independently of FBXL19 (Stably Induced, green), and those that rely on FBXL19 for their induction (Responsive, blue). The boxplots display the median, 25th and 75th quartile. P-values: Student's t-test.

(E) A density plot illustrating the distances to nearest TSS for ATAC-seq peaks interacting with FBXL19-bound genes that are not induced (dashed-black), induced independently of FBXL19 (Stably Induced, green), and those that rely on FBXL19 for their induction (Responsive, blue).

(F) As in E, but distances are illustrated between gene promoters and their interacting distal sites.

(G) A metaplot of RNAPolII ChIP-seq enrichment (43) centred at ATAC-seq peaks interacting with either not induced (dashed-black), stably induced (green) or FBXL19-responsive (blue) FBXL19-bound gene promoters.

(H) Boxplots showing 4su-RNA-seq (24) enrichment in ESCs at active enhancers (red) (25) and at CDK8-occupied sites interacting with promoters of FBXL19-occupied FBXL19-responsive genes (blue), genes induced independently of FBXL19 binding (green) or genes that are not induced upon RA-differentiation (grey). P-values: Student's t-test.

(I) As in G but for FBXL19-FS2 ChIP-seq (24). Promoter-interacting ATAC-seq peaks were divided into those that overlap an annotated TSS (TSS, left) and those without such an overlap (nonTSS, right).

(J) A metaplot of CDK8 ChIP-seq enrichment (43) centred at ATAC-seq peaks interacting with FBXL19-responsive gene promoters. ChIP-seq signal is compared between untreated (dark-blue) and tamoxifen-treated (light-blue) *FBXL19-CxxC*$^{fl/fl}$ ESCs.

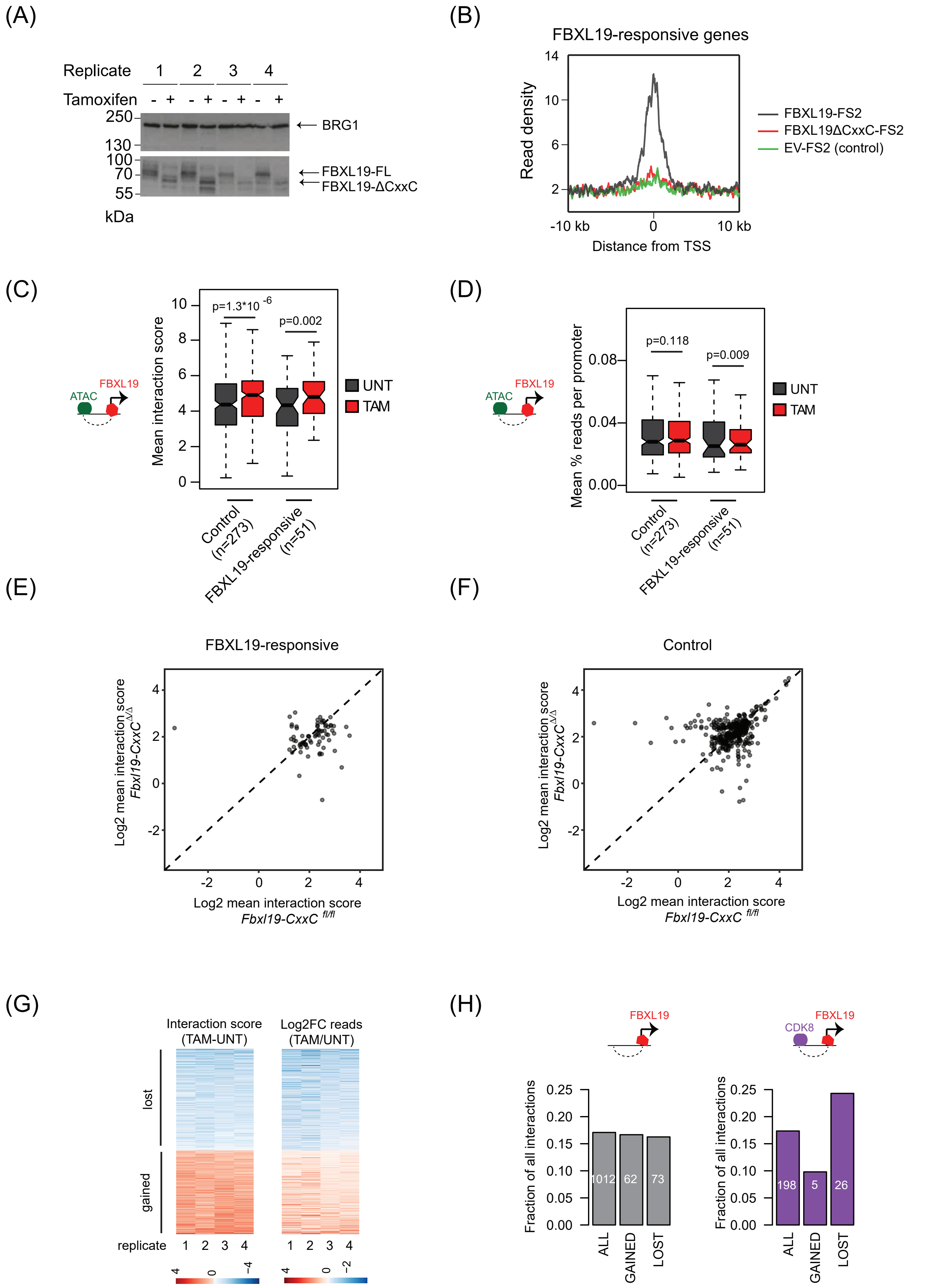

Feldmann et al_Fig.S2

Figure S2. Related to Figure 2

(A) A western blot illustrating the deletion of *Fbxl19 CxxC* domain in four biological replicates. BRG1 was used as loading control.

(B) A metaplot of an FS2-tagged wildtype (FBXL19-FS2), CxxC-domain deficient FBXL19 protein (FBXL19dCxxC-FS2) or empty vector control (EV-FS2) ChIP-seq enrichments (24) centred at promoters of FBXL19-responsive genes.

(C) A boxplot showing the Chicago interaction scores for interactions between ATAC-seq peaks not occupied by CDK8 and FBXL19-responsive or control gene promoters. The boxplots show mean interaction score between test promoters and CDK8-occupied sites in untreated (UNT) or tamoxifen-treated (TAM) *Fbxl19-CxxC*$^{fl/fl}$ ESCs. The control sites correspond to FBXL19-bound, but not FBXL19-responsive, genes. The number of interactions quantified is indicated below the boxplots. P-values: paired t-test.

(D) As in B, but showing normalized read counts.

(E) A scatter plot comparing the interaction scores between untreated and tamoxifen-treated *Fbxl19-CxxC*$^{fl/fl}$ ESCs for interactions formed by FBXL19-responsive gene promoters with CDK8-occupied distal sites.

(F) As in D but for interactions formed by FBXL19-bound, but not FBXL19-responsive, genes.

(G) Heatmaps illustrating the difference in interaction score (left) and log2 fold change in normalized read counts (right) for interactions that are either lost or gained in tamoxifen-treated *Fbxl19-CxxC*$^{fl/fl}$ in relation to untreated ESCs.

(H) A bar chart illustrating the fraction of interactions detected in the Capture-C data that are formed between FBXL19-responsive gene promoters and any other (grey, left) or CDK8-occupied (purple, right) sites in the genome for all interactions, interactions that are gained in tamoxifen-treated (TAM) *Fbxl19-CxxC*$^{fl/fl}$ ESCs and interactions that are lost in tamoxifen-treated (TAM) *Fbxl19-CxxC*$^{fl/fl}$ ESCs. The number of assessed interactions is indicated inside the bars.

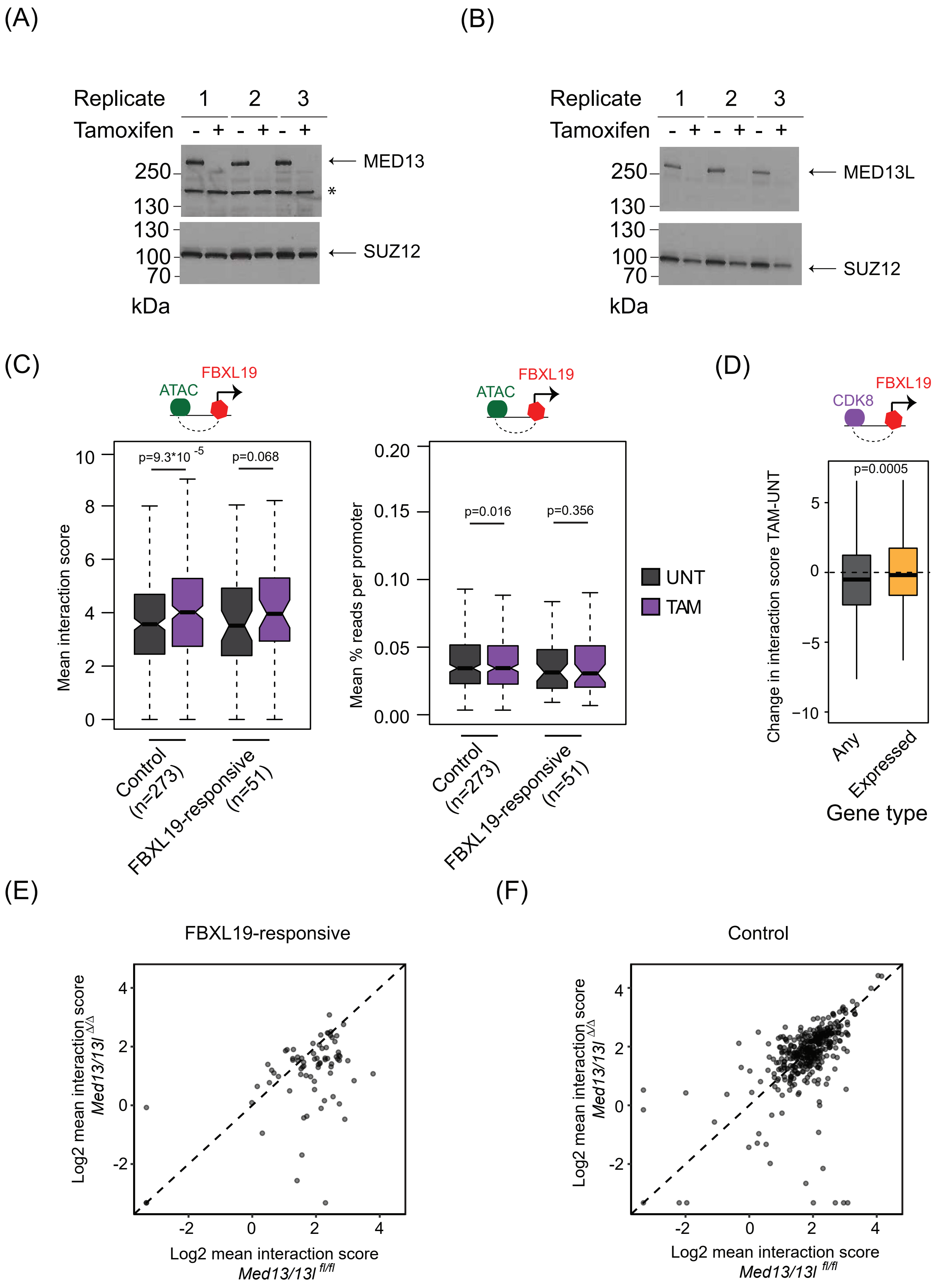

Feldmann et al_Fig.S3

Figure S3. Related to Figure 3

(A) A western blot illustrating the deletion of MED13 in three biological replicates. SUZ12 was used as loading control. The asterisk indicates an unspecific band.

(B) As in (A) but for MED13L.

(C) A boxplot showing the Chicago interaction scores (left) or mean normalized read counts (right) for interactions between ATAC-seq peaks not occupied by CDK8 and FBXL19-responsive or control gene promoters. The boxplots show mean interaction score between test promoters and CDK8-occupied sites in untreated (UNT) or tamoxifen-treated (TAM) *Med13/13l*$^{fl/fl}$ ESCs. The control sites correspond to FBXL19-bound, but not FBXL19-responsive, genes. The number of interactions quantified is indicated below the boxplots. P-values: paired t-test.

(D) A boxplot illustrating the difference in mean interaction scores between *Med13/13l*$^{fl/fl}$ (UNT) and *Med13/13l*$^{\Delta/\Delta}$ (TAM) ESCs for interactions formed by promoters of transcriptionally active (expressed, orange) or other genes (dark-grey) with CDK8-occupied distal sites. P-value: Student's t-test.

(E) A scatter plot comparing the interaction scores between untreated and tamoxifen-treated *Med13/13l*$^{fl/fl}$ ESCs for interactions formed by FBXL19-responsive gene promoters with CDK8-occupied distal sites.

(F) As in E but for interactions formed by FBXL19-bound, but not FBXL19-responsive, genes.

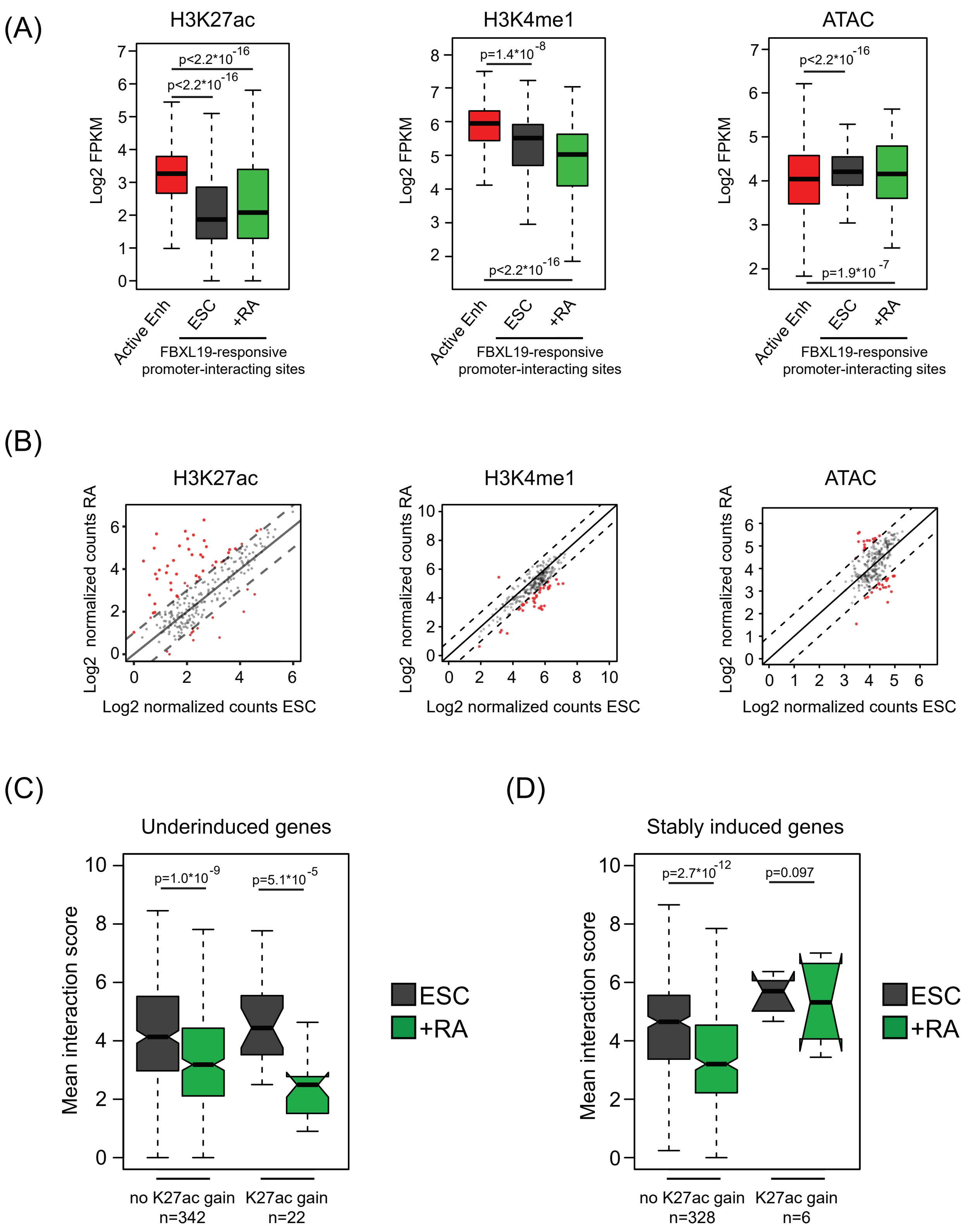

Feldmann et al_Fig.S4

Figure S4. Related to Figure 4

(A) Boxplots showing H3K27ac, H3K4me1 and ATAC-seq enrichment at active ESC enhancers (red) (25) and at CDK8-occupied sites interacting with promoters of FBXL19-responsive genes before (grey) or after (green) RA-differentiation. P-values: Student's t-test.

(B) Scatterplots comparing H3K27ac, H3K4me1 and ATAC-seq enrichment between untreated and retinoic-acid treated ESCs (RA). Shown are only accessible, CDK8-occupied promoter distal sites, which interact with FBXL19-responsive genes. Sites with a gain or loss of at least 2-fold are highlighted in red. The dashed lines indicate the 2-fold cut-off applied to determine whether there was a gain or loss of the respective feature and the solid line corresponds to the diagonal.

(C) A boxplot showing the Chicago interaction scores for interactions between CDK8-occupied accessible sites and underinduced gene promoters. The boxplots show mean interaction score between test promoters and CDK8-occupied sites that acquire H3K27ac or maintain their levels upon differentiation in untreated (ESC) or retinoic acid-treated (RA) *Fbxl19-CxxC*$^{fl/fl}$ ESCs. The number of interactions quantified is indicated below the boxplots. P-values: Student's t-test.

(D) As in A, but scores are shown for genes that are induced independently of FBXL19.

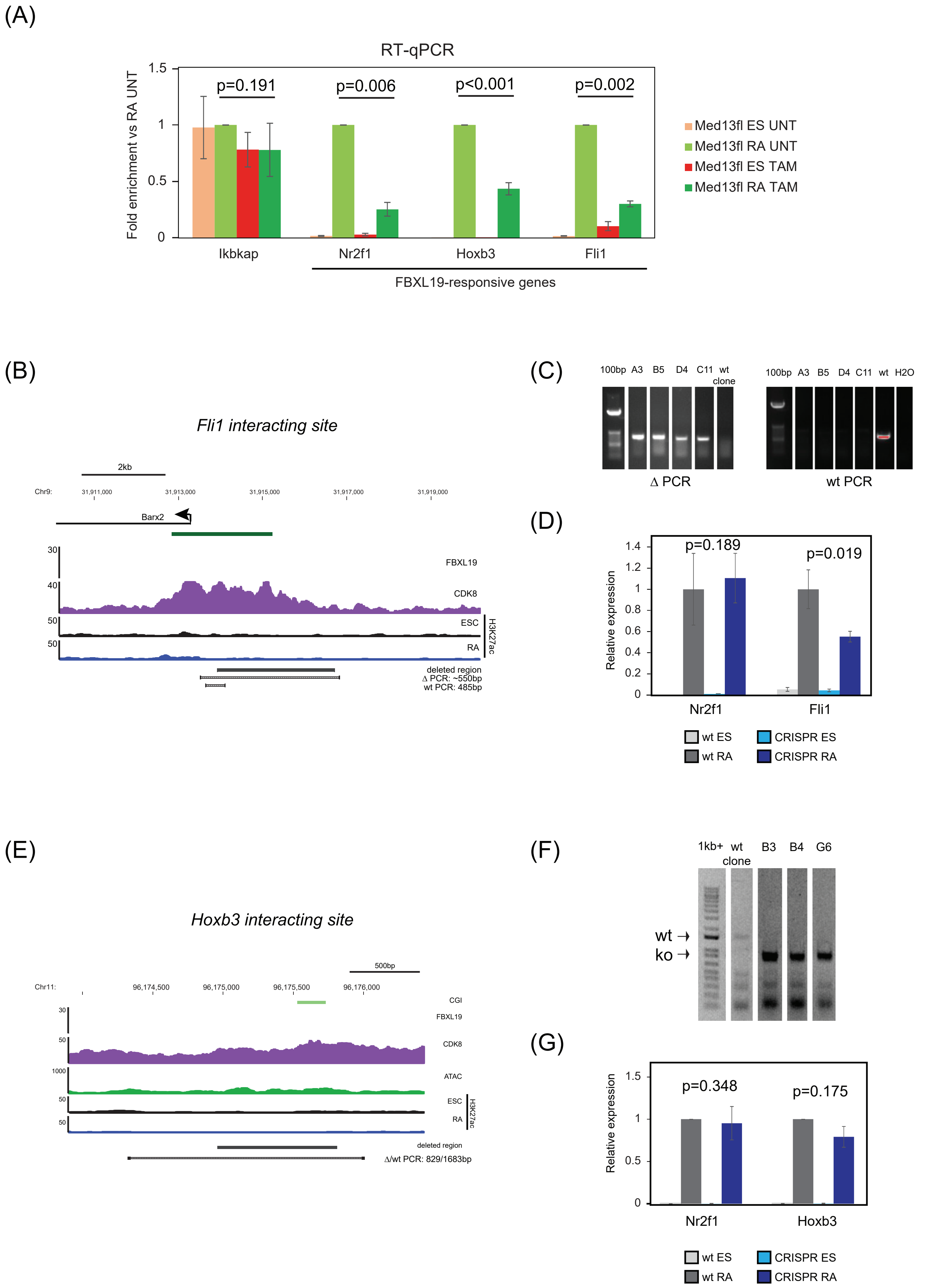

Feldmann et al_Fig.S5

Figure S5. Related to Figure 5

(A) Relative mRNA-levels for *Ikbkap*, *Bcor*, *Nr2f1*, *Hoxb3* and *Fli1* in untreated (UNT) and tamoxifen-treated (TAM) *Med13/13l*$^{fl/fl}$ ESCs prior (ES) and after (RA) RA-induced differentiation as determined by RT-qPCR. Shown is the mean signal normalised internally to a control gene and to expression in tamoxifen-untreated differentiated *Med13/13l*$^{fl/fl}$ ESCs (Med13fl RA UNT). Error bars indicate standard error of mean across 4 biological replicates. P-values: Student's t-test.

(B) Screenshot of *Fli1* interacting site with deleted region and amplified PCR fragments for screening indicated below. ΔPCR: PCR to detect deletion, wt PCR: PCR to detect intact alleles.

(C) Screening for clones with a homozygous deletion (left) and a wildtype (right) allele of the *Fli1* interacting site. DNA ladder used is 1kb Plus DNA ladder (Thermo Fischer Scientific). The gel image represents bands from a single screening gel, rearranged to show clones used in this study.

(D) Relative mRNA-levels of *Nr2f1* and *Fli1* in untreated and RA-treated wildtype ESC clones (n=2) and in ESC clones in which the *Fli1* promoter interacting site has been deleted (n=4) as determined by RT-qPCR. Shown is the mean signal normalised internally to a control gene and to expression in wildtype clones treated by retinoic acid (RA). Error bars indicate standard error of mean across 4 biological and 3 technical replicates. P-values: Student's t-test.

(E) Same as in B but for the *Hoxb3* interacting site.

(F) Same as in C, but for the *Hoxb3* interacting site.

(G) Same as in D, but for the *Hoxb3* interacting site deletion clones (n=3) and corresponding wildtype clones (n=4).

Supplementary Tables

Table S1. Capture-C Hybridization probes

Table S2. Annotated Promoter Interacting Fragments (This study)

Table S3. Replicate Correlations Capture-C
